# Combined impact of glucose variability and mineral disorders on mortality and severe cardiorenal events in critically ill CKD patients: a multicenter cohort study

**DOI:** 10.3389/fmed.2026.1826900

**Published:** 2026-07-08

**Authors:** Yingyun Peng, Yuxi Jia, Hui Xu, Jiali Yao, Lijuan Jiang

**Affiliations:** 1 Health Management Center, Affiliated Jinhua Hospital, Zhejiang University School of Medicine, Jinhua, China; 2 Tongji Medical College, Huazhong University of Science and Technology, Wuhan, China; 3 Department of Stomatology, Affiliated Jinhua Hospital, Zhejiang University School of Medicine, Jinhua Municipal Central Hospital, Jinhua, China; 4 Department of Critical Care Medicine, Jinhua Hospital Affiliated to Zhejiang University, Jinhua, Zhejiang, China; 5 Division of General Practice, People’s Hospital of Chongqing Hechuan, Chongqing, China

**Keywords:** chronic kidney disease, glucose variability, mineral disorders, mineral metabolism, mortality risk

## Abstract

**Introduction:**

Elderly critically ill ICU patients with CKD face high mortality. The independent and combined prognostic roles of glycemic variability (Glucose CV) and calcium-phosphate metabolic disorders in this population remain unconfirmed.

**Methods:**

This retrospective study contained three cohorts: MIMIC-IV (*n* = 1,319, development), multicenter eICU (*n* = 996, validation), and Chinese hospital cohort (*n* = 1,069, clinical validation). Multivariable Cox regression and meta-analysis were applied.

**Results:**

Elevated Glucose CV independently predicted 28- and 90-day mortality with predominant early risk. Concurrent hypocalcemia and hyperphosphatemia indicated the poorest prognosis, and high GV produced synergistic harmful effects in this subgroup. All results were verified by external cohorts.

**Discussion:**

Targeted glycemic variability management and calcium-phosphate homeostasis correction may effectively improve survival of elderly critically ill CKD patients.

## Introduction

Critically ill elderly patients with chronic kidney disease (CKD) represent a particularly vulnerable population facing a disproportionately high burden of in-hospital mortality and long-term adverse outcomes, imposing significant strain on global healthcare systems (1, 2). The pathophysiology of CKD involves a complex interplay of metabolic derangements, including systemic inflammation, oxidative stress, and endocrine dysregulation, which are further exacerbated during acute critical illness (3). This convergence of chronic organ dysfunction and acute physiological stress creates a high-risk clinical scenario where traditional, single-system risk assessment paradigms may be insufficient to capture the integrated metabolic disturbances driving poor prognosis. Current management strategies in the intensive care unit (ICU) often focus on stabilizing individual organ systems or correcting isolated metabolic parameters, such as hyperglycemia or electrolyte imbalances. However, this compartmentalized approach may overlook the synergistic deleterious effects of concurrent metabolic pathway disruptions, which could be pivotal in determining patient trajectories.

Among the myriad metabolic disturbances in critically ill CKD patients, dysglycemia and bone-mineral disorders are two prominent and potentially interconnected pathways. Glucose metabolism instability, often quantified as glucose variability (GV), has emerged as a significant prognostic factor beyond average glucose levels in various critically ill populations (4). Acute fluctuations in blood glucose can induce oxidative stress, endothelial dysfunction, and sympathetic activation, potentially exacerbating organ injury (5). Concurrently, CKD-Mineral and Bone Disorder (CKD-MBD) is a hallmark complication of renal dysfunction, characterized by abnormalities in calcium, phosphate, parathyroid hormone (PTH), and vitamin D metabolism (6). These disturbances are not confined to skeletal health but are intrinsically linked to vascular calcification and cardiovascular morbidity, which are leading causes of mortality in this population (7). Prior research has independently established hyperglycemia/insulin resistance and CKD-MBD as key contributors to adverse outcomes. Notably, emerging evidence suggests a biological crosstalk between glucose and mineral metabolism. For instance, bone acts as an endocrine organ secreting factors like osteocalcin, which can influence insulin sensitivity, while insulin resistance and inflammatory states can adversely affect bone remodeling (8). This established yet underexplored interconnection highlights a critical knowledge gap: the combined prognostic impact of acute glucose instability and specific bone-mineral disturbance phenotypes in the high-stakes setting of critical illness among elderly CKD patients.

Despite the recognized individual risks, a significant research gap persists regarding the integrated prognostic role of these interacting metabolic pathways. Most studies have evaluated GV or bone-mineral parameters in isolation, often in general ICU populations or stable CKD cohorts, neglecting their potential synergistic effect during acute decompensation in elderly CKD patients. Furthermore, the prognostic value of specific, readily identifiable acute bone-mineral disturbance phenotypes (e.g., isolated hypocalcemia, hyperphosphatemia, or their combination) in the early ICU phase remains poorly characterized. The temporal dynamics of GV-associated risk and its interaction with concurrent mineral imbalances are also unclear. Filling this gap is necessary to move beyond static, single-metric assessments towards a more holistic, pathophysiologically-informed risk stratification model that can identify patients at the highest risk for mortality and severe cardiorenal complications.

To address these questions, this study employs a robust methodological approach leveraging large-scale, real-world clinical data. We conducted a retrospective observational cohort study utilizing two independent, publicly available ICU databases: the Medical Information Mart for Intensive Care IV (MIMIC-IV) database for model derivation and the eICU Collaborative Research Database (eICU-CRD) for external validation. This dual-cohort design enhances the generalizability and robustness of the findings by testing associations across diverse clinical settings and patient populations. The use of these granular databases allows for the detailed capture of dynamic parameters such as serial glucose measurements for calculating GV (e.g., coefficient of variation) and early ICU laboratory values defining bone-mineral phenotypes, facilitating a comprehensive analysis of their early and time-varying associations with outcomes.

The primary objectives of this investigation are threefold. First, to evaluate the independent and time-varying association of early ICU glucose variability with short-term (28-day) and medium-term (90-day) all-cause mortality in critically ill elderly patients with pre-existing CKD. Second, to assess the prognostic value of acute bone-mineral disturbance phenotypes, identified from initial ICU laboratory assessments, for predicting mortality and composite cardiorenal outcomes. Third, and most critically, to investigate the potential interaction between glucose variability and adverse bone-mineral phenotypes on the risks of mortality and major cardiorenal events, testing the hypothesis that their co-existence confers a synergistic risk greater than the sum of their individual effects. By elucidating these relationships, this study aims to provide evidence for an integrated metabolic risk model that could inform early targeted monitoring and multimodal intervention strategies in this vulnerable patient group.

## Methods

This study is a retrospective observational cohort study. The development cohort was derived from the MIMIC-IV database (v3.1), which includes critically ill patients admitted to the Beth Israel Deaconess Medical Center between 2008 and 2019. The validation cohort was obtained from the eICU Collaborative Research Database (v2.0), which contains patient data from ICUs of 208 hospitals in the United States between 2014 and 2015. The authors of this study obtained data access permissions through the Collaborative Institutional Training Initiative (CITI) certification (certification number: 68028865), and both databases have removed identifying information, exempting the requirement for informed consent. Additionally, data from ICU patients at Zhejiang Jinhua Hospital from 2015 to 2025 were collected, and the study was approved by the Ethics Committee of Zhejiang Jinhua Hospital (No. 2025–186).

### Study population

We used Navicat Premium to identify the study cohort in both databases using a conceptually consistent but moderately adjusted operational definition strategy to assess the robustness of the conclusions. The specific inclusion and exclusion criteria are as follows: (1) Age ≥ 65 years; (2) First ICU admission; (3) Chronic kidney disease (CKD) (meeting either of the two conditions): a. Relevant ICD diagnostic codes or diagnostic characters; b. The most recent eGFR < 60 mL/min/1.73m^2^ before ICU admission (the moderately relaxed standard in eICU is: eGFR < 60 before and after admission). (4) Within 24 h of ICU admission (moderately relaxed to 48 h in eICU): at least 2 blood glucose records (to calculate GV) and at least 1 record each of triglycerides, calcium, and phosphorus.

Exclusion Criteria: (1) ICU stay too short (<6 h); (2) Extreme hyperglycemic crisis (DKA); (3) Received renal replacement therapy (RRT) within 24 h of ICU admission (moderately relaxed to 48 h in eICU). (4) Kidney transplant patients. Based on the actual situation, the clinical patient data were selected according to the same standards as those for eICU.

### Data

Demographic data of patients (age, sex), triglycerides, blood glucose, calcium, phosphorus, comorbidities (mainly including diabetes, heart failure), disease severity scores (SOFA used in MIMIC-IV, APACHE IV used in eICU), comorbidity burden (Charlson Comorbidity Index, diabetes status), and early treatment conditions (such as insulin use within 24/48 h, use of vasoactive drugs, and mechanical ventilation status) were extracted.

The main exposure variables were measured within 24 h (48 h in eICU). TyG = ln[fasting triglycerides (mg/dL) × fasting glucose (mg/dL) / 2]. Blood glucose variability Glucose Coefficient of Variation (GV) = standard deviation/mean × 100%, calculated using all blood glucose values within the time window. Blood glucose measurements were obtained from routine point-of-care (POC) capillary glucose testing per standard ICU practice. Continuous glucose monitoring (CGM) data were not available in any of the three cohorts.

Acute mineral metabolism disorder phenotypes: defined based on the first calcium and phosphorus measurements within the time window as “pure hypocalcemia,” “pure hyperphosphatemia,” “hypocalcemia combined with hyperphosphatemia,” and “normal.” Due to the different coverage rates of calcium indicators in the three cohorts, in MIMIC-IV and the actual clinical cohort, the ion calcium level was selected (low calcium was defined as < 1.12 mmol/L), while in the eICU cohort, the total calcium level was chosen (low calcium was defined as < 2.10 mmol/L). Albumin correction was not applied to total calcium because albumin data were missing in >85% of eICU patients. This approach is consistent with recent evidence and a 2026 joint position statement (EFLM/IOF/IFCC) recommending against routine albumin-adjusted calcium reporting in CKD and critically ill populations (see Discussion) (9–11).

Primary Outcome Indicators: MIMIC-IV for 28-day mortality; eICU for in-hospital mortality. Secondary outcome indicators: 90-day mortality, cardiovascular/kidney-related outcomes (new RRT and shock).

In the eICU-CRD and actual clinical data, we verified the independent effect of Glucose CV after adjusting for TyG and clinical variables (regarding in-hospital mortality), as well as whether the interaction direction between Glucose CV and mineral metabolism phenotypes was consistent with that of MIMIC. Due to the lack of post-discharge follow-up in eICU and actual clinical data, secondary outcome indicators included long-term mortality and complex cardiovascular and renal outcomes, which were only evaluated in MIMIC-IV.

### Statistical analysis

All data organization and statistical analyses were performed using R software (version 4.3.1). Extreme values of continuous variables were winsorized (truncated at the 1st and 99th percentiles). The handling of missing covariates preset rules: variables with a missing rate <5% were analyzed using complete case analysis; variables with a missing rate of 5–20% were handled using multiple imputation chained equations (MICE), where the imputation model included all regression variables and outcome indicators, 5 imputed datasets were generated using predictive mean matching (PMM), and results were pooled to Rubin’s rules; variables with a missing rate >20% were excluded from the main model and addressed as a limitation in the discussion. In the MIMIC-IV, the missing rate of calcium was 17.5% (231/1319), falling within the 5–20% range, thus ICE imputation was used for the main analysis. To verify the robustness of the imputation, all analyses were repeated in the complete calcium subset without imputation (n = 1,088). Continuous variables were tested for normality using the Kolmogorov–Smirnov test, and based on their skewed distribution characteristics, median (interquartile range) was used for description; categorical variables were described using frequency (percentage). Comparisons between groups for continuous variables conforming to normal distribution were performed using t-tests, non-normally distributed continuous variables were analyzed using Mann–Whitney U tests, and categorical variables were analyzed using chi-square tests or Fisher’s exact tests (when expected frequency <5). Standardized mean differences (SMD) were used to assess baseline differences between cohorts.

Kaplan–Meier curves were used to evaluate survival differences between different Glucose CV groups or mineral metabolism disorder groups, and Log-rank tests were used to compare differences between groups. Restricted cubic splines were used to assess the correlation between GV and mortality risk in different cohorts, and Wald tests were used to assess the significance of nonlinear terms. Cox proportional hazards regression was used to assess the correlation between Glucose CV, mineral metabolism phenotypes, and short-term mortality, adjusting for confounding factors such as age, sex, and further adjusting for disease severity, comorbidities, treatment variables, and other confounding factors, using Schoenfeld residual tests to check for violations of the proportional hazards assumption. Stratified Cox models were used for variables that violated the assumption, using time interaction terms in the covariate model to handle time-varying effects, or using segmented Cox models: segmented by time (7, 14, 21 days).

Subgroup analyses were stratified by mineral metabolism phenotypes, and interaction tests primarily assessed the interaction effect of GV × mineral metabolism phenotypes, using forest plots to display subgroup-specific effects. The main analysis of MIMIC was repeated in the eICU cohort and actual clinical data using logistic regression analysis, comparing effect sizes and consistency with the direction of the main cohort. The two cohorts were combined, and fixed-effect meta-analysis was performed on log(HR/OR) using inverse variance weighting, with *I*^2^ and *Q* tests used to assess quality.

## Results

**Figure 1:**
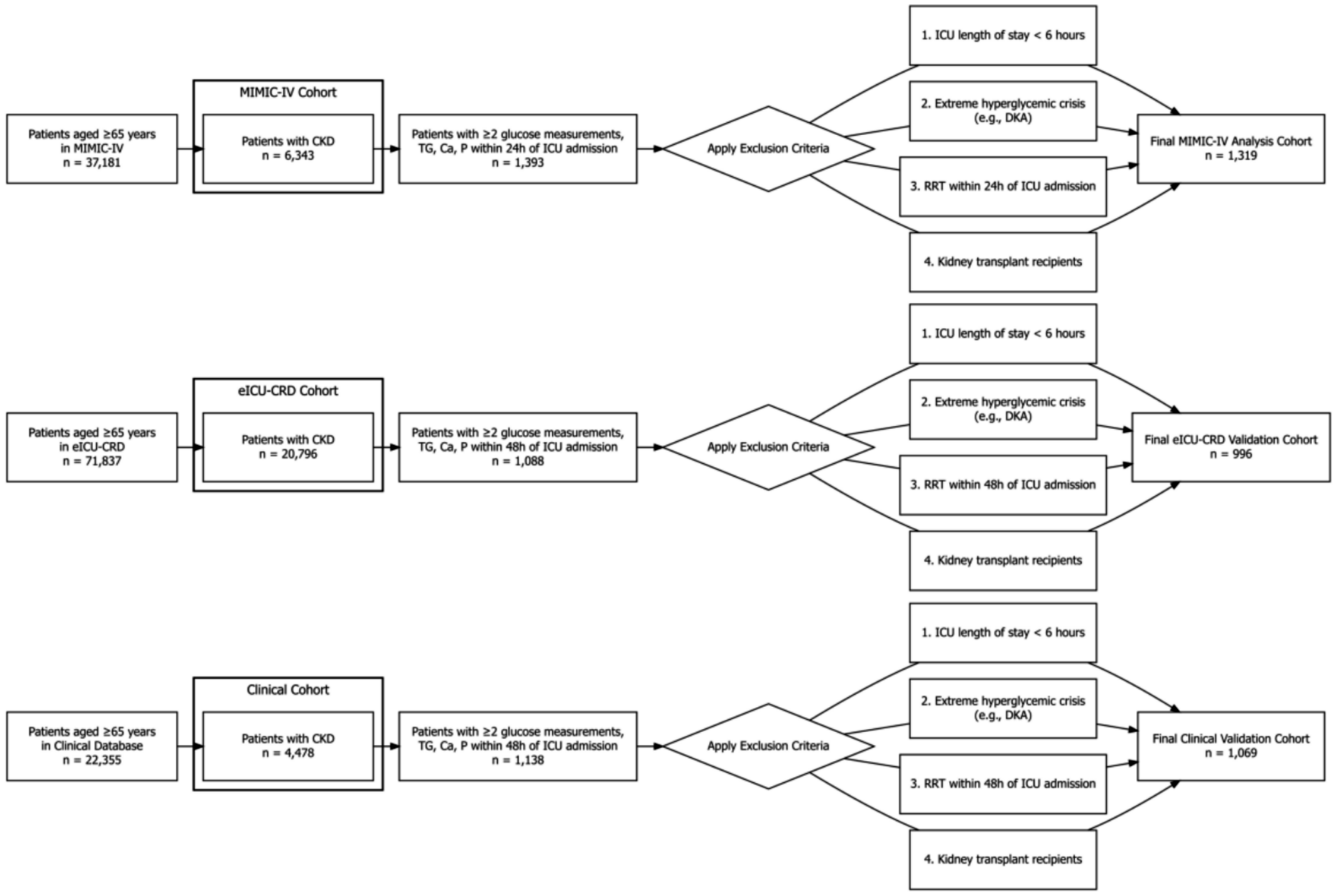
Flowchart of participant selection in the MIMIC-IV, eICU-CRD and clinical cohorts.

This study included a total of 1,319 individuals in the main cohort of MIMIC-IV, 996 individuals in the validation cohort of eICU-CRD, and 1,069 individuals in the clinical cohort (Figure 1). The baseline characteristics (Table 1) of the three cohorts showed that the clinical validation cohort and the discovery cohort (MIMIC-IV) were highly similar in demographic and clinical characteristics (SMD range 0.012–0.215), indicating good comparability between the two cohorts. The multicenter eICU cohort showed moderate differences (SMD 0.2–0.6) (Supplementary Figure S1), reflecting heterogeneity between single-center and multicenter practices.

**Table 1:** Baseline characteristics and standardized mean differences.

**Variable**	Groups	SMD1*	**SMD2†**	**MIMIC-IV** **(N = 1,319)**	**eICU-CRD** **(N = 996)**	**Clinical cohort** **(N = 1,069)**
Age		0.068	0.071	76.28 [70.48, 83.26]	76.00 [70.00, 83.00]	75.81 [70.00, 82.48]
Calcium (mmol/L)		—	0.215	1.10 [1.04, 1.16]	2.10 [1.25, 2.25]	1.11 [1.04, 1.17]
Phosphate (mmol/L)		0.755	0.101	1.68 [1.32, 2.13]	1.26 [1.00, 1.58]	1.58 [1.26, 2.07]
Glucose CV		−0.234	0.088	0.15 [0.08, 0.26]	0.20 [0.12, 0.30]	0.17 [0.09, 0.28]
TyG		−0.578	0.101	7.79 [7.07, 9.21]	8.97 [8.53, 9.46]	8.28 [7.24, 9.32]
Gender	0	0.146	0.045	832 (63.1)	557 (55.9)	418 (39.1)
	1			487 (36.9)	439 (44.1)	651 (60.9)
Diabetes	0	0.265	0.042	689 (52.2)	649 (65.2)	581 (54.3)
	1			630 (47.8)	347 (34.8)	488 (45.7)
Calcium-phosphate phenotype	Normal	0.43	0.117	334 (25.3)	364 (36.5)	242 (22.6)
	Hypocalcemia			204 (15.5)	287 (28.8)	220 (20.6)
	Hyperphosphatemia			539 (40.9)	138 (13.9)	247 (23.1)
	Hypocalcemia and Hyperphosphatemia			242 (18.3)	207 (20.8)	360 (33.7)
Vasopressor use	0	0.285	0.012	827 (62.7)	754 (75.7)	664 (62.1)
	1			492 (37.3)	242 (24.3)	405 (37.9)
Mechanical ventilation	0	0.164	0.119	786 (59.6)	672 (67.5)	574 (53.7)
	1			533 (40.4)	324 (32.5)	495 (46.3)
Insulin use	0	0.517	0.042	930 (70.5)	900 (90.4)	774 (72.4)
	1			389 (29.5)	96 (9.6)	295 (27.6)

**Cohort**	**Variable**	Value
MIMIC-IV (*N* = 1,319)	SOFA Score	3.0 [0.0, 5.0]
	Charlson Comorbidity Index	8.0 [7.0, 10.0]
	28-day Mortality	-
	0	864 (65.5)
	1	455 (34.5)
	RRT within 7 days	-
	0	1,097 (83.2)
	1	222 (16.8)
	Shock > 48 h	-
	0	1,070 (81.1)
	1	249 (18.9)
eICU-CRD (*N* = 996)		N = 996
	APACHE IV Score	70.50 [56.0, 89.0]
	Hospital Mortality	-
	0	787 (79.0)
	1	209 (21.0)
Clinical (*N* = 1,069)		
	SOFA Score	4.0 [0.0, 7.0]
	Charlson Comorbidity Index	7.0 [5.0, 10.0]
	Hospital Mortality	-
	0	721 (67.4)
	1	348 (32.6)

CV: Coefficient of variation; Continuous variables: median [IQR]; Categorical: n (%). SMD: Standardized mean difference between MIMIC-IV (discovery) and other cohorts. SMD1*: MIMIC-IV vs eICU-CRD; SMD2†: MIMIC-IV vs Clinical Validation. SMD < 0.1 = Negligible difference; 0.1–0.3 = Small difference. Clinical cohort showed minimal baseline imbalance with discovery cohort.

Patients in the validation cohort had relatively milder disease severity, as indicated by significantly lower rates of vasopressor use (24.3% vs. 37.3%), mechanical ventilation (32.5% vs. 40.4%), and insulin use (9.6% vs. 29.5%) compared to the discovery cohort (all SMD > 0.1). The 28-day mortality rate in MIMIC-IV (34.5%) was also higher than the in-hospital mortality rate in eICU (21.0%). Core metabolic indicators also showed significant differences: the TyG index was higher in the validation cohort (median 8.97 vs. 7.79, SMD = −0.578), and the blood glucose variability coefficient was larger (0.20 vs. 0.15, SMD = −0.234). The distribution of bone mineral metabolism disorder phenotypes was markedly different between the two cohorts, with a significantly lower proportion of hyperphosphatemia phenotype in the validation cohort (13.9% vs. 40.9%), reflecting the differences between single-center and multi-center laboratories. The significant differences in calcium measurement levels between the two cohorts were due to the different calcium indicators used. These baseline differences confirm that the validation process of this study was conducted in independent medical environments with different patient characteristics, treatment patterns, and testing systems, representing a rigorous external validation. Random effects meta-analysis summarized the cohort-specific estimates from MIMIC-IV and eICU (Supplementary Figure S2), with a combined effect size of 0.3 (95% CI 0.08–0.55, *p* = 0.008), with no evidence of heterogeneity between cohorts (*I* = 0%, *P* heterogeneity = 0.85).

**Figure 2:**
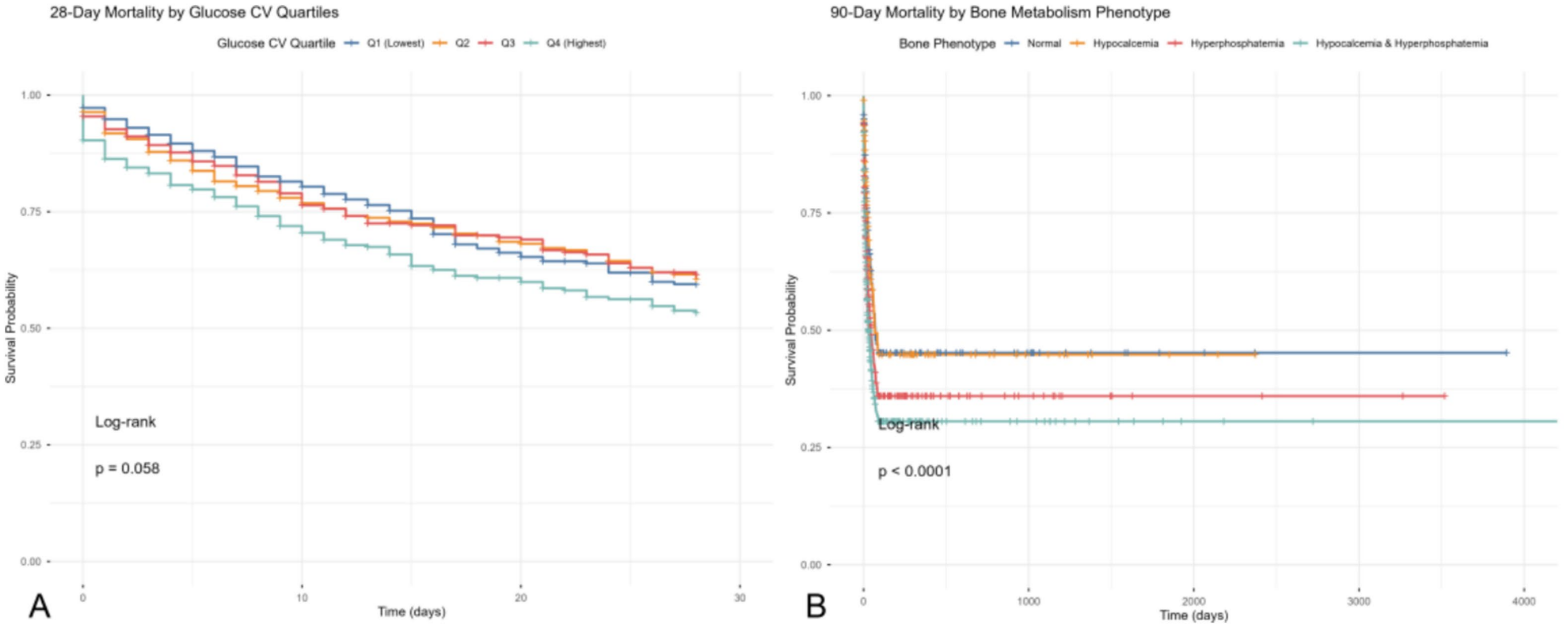
Kaplan–Meier survival curve. **(A)** Patients with greater glucose variability (Q4 group) had a lower probability of survival within 28 days, but the trend was only marginally significant (*p* = 0.058). **(B)** Abnormal bone mineral metabolism was significantly associated with 90-day mortality risk, especially hypocalcemia and hyperphosphatemia.

To explore the correlation between Glucose CV and mortality, blood glucose was grouped by quartiles (with Q1 as reference), finding that patients with high blood glucose variability (Q4 group) may have lower survival probabilities within 28 days, although this trend was only marginally significant statistically (Figure 2A). To enhance clinical interpretability, we calculated the absolute 28-day mortality rates by glucose variability quartile in the MIMIC-IV cohort. The mortality rates were 33.0, 32.7, 32.2, and 40.0% for Q1 through Q4, respectively (Supplementary Table S1), corresponding to an absolute risk difference of 7.0 percentage points between Q4 and Q1. Mineral metabolism abnormal phenotype (especially the hypocalcemia combined with hyperphosphatemia group) was significantly associated with 90-day mortality risk (Figure 2B), suggesting that mineral metabolism disorders are important factors affecting mid-term prognosis in elderly critically ill CKD patients.

**Figure 3:**
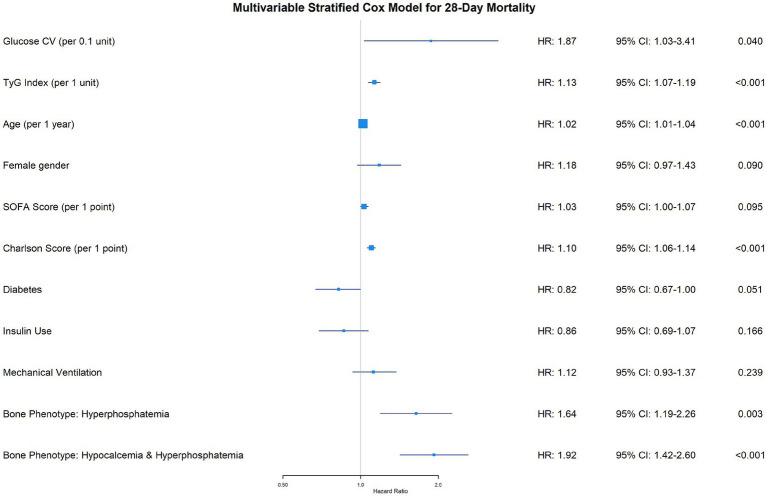
Full multivariable stratified Cox model for 28-day mortality (MIMIC-IV). Adjusted for all covariates shown. *p < 0.05, **p < 0.01, ***p < 0.001. Horizontal lines: 95% CI. The complete stratified Cox model confirms glucose CV (HR 1.87*) alongside TyG index (HR 1.13***) and combined calcium-phosphate phenotype (HR 1.92***).

In the MIMIC-IV cohort, in a stratified Cox model fully adjusted for confounding factors such as age, sex, severity of illness score (SOFA), and comorbidities, glucose variability (Glucose CV) independently predicted 28-day mortality risk (HR = 1.87, 95% CI 1.03–3.41, *p* = 0.040) and 90-day mortality risk (HR = 1.80, 95% CI 1.06–3.08, *p* = 0.031) (Table 2 and Figure 3). The TyG index also showed strong associations (28-day HR 1.13, *p* < 0.001; 90-day HR 1.9, *p* < 0.001). Some variables violated the proportional hazards assumption (Glucose CV *p* = 0.004, insulin use *p* = 0.0076, etc.; Supplementary Table S3). Stratified Cox (by sex, insulin use, mechanical ventilation) and time-dependent Cox (Glucose CV, TyG) models effectively addressed the issue, with global tests passing. The time-dependent Cox model further revealed (Supplementary Table S2) that the risk effect of Glucose CV exhibited an early high-risk, later attenuation pattern (Supplementary Figure S2, early HR = 3.84, 95% CI 1.45–10.15, p = 0.007; time interaction term *p* = 0.065), suggesting that this effect is primarily concentrated in the acute phase of the disease. The C-index for the stratified Cox models was 0.625 (28-day) and 0.645 (90-day), respectively. The combined low calcium-hyperphosphatemia calcium-phosphate phenotype had the highest risk (Table 3): 28 days HR 1.92 (1.42–2.60, *p* < 0.001); 90 days HR 1.76 (1.6–2.26, *p* < 0.001).

**Table 2:** Multivariable associations with mortality in MIMIC-IV cohort.

**Variable**	**28-day Mortality** **(*n* = 1,319, 35%)**		**90-day Mortality** **(*n* = 1,319, 46%)**	
	**HR (95% CI)**	***p* value**	**HR (95% CI)**	***p* value**
Glucose CV (per 0.1 unit)	1.87 (1.03–3.41)	0.04*	1.80 (1.06–3.08)	0.031*
TyG index (per 1 unit)	1.13 (1.07–1.19)	<0.001***	1.09 (1.04–1.14)	0.0004***
Age (per year)	1.02 (1.01–1.03)	0.002**	1.02 (1.01–1.03)	0.0002***
SOFA score	1.04 (1.00–1.07)	0.034*	1.02 (0.99–1.05)	0.154
Charlson score	1.10 (1.06–1.13)	<0.001***	1.07 (1.03–1.11)	0.0002***
Diabetes	0.82 (0.67–1.00).	0.052	0.82 (0.69–0.97)	0.024*
Model diagnostics:	–	–	–	–
C-index	0.625 (0.015)	–	0.645 (0.012)	–
Events/Total (%)	459/1319 (34.8%)	–	613/1319 (46.5%)	–

*p < 0.05, **p < 0.01, ***p < 0.001.

**Table 3:** Multivariable associations of glucose variability with mortality across cohorts and meta-analysis.

**Cohort**	**Exposure contrast**	**OR (95% CI)**	***p* value**
MIMIC-IV (*n* = 1,319)	Q4 vs. Q1	1.36 (1.05–1.77)	0.02
eICU-CRD (*n* = 996)	Q4 vs. Q1	1.44 (0.83–2.49)	0.192
Clinical (*n* = 1,069)	Q4 vs. Q1	4.09 (1.45–12.8)	0.0105
Pooled Meta-analysis (primary)	Q4 vs. Q1	1.44 (1.15–1.82)	0.0018
Sensitivity: In-hospital only	Q4 vs. Q1	1.47 (1.11–1.93)	0.0066

Outcome: In-hospital/28-day mortality (adjusted for age, sex, diabetes, insulin use, vasopressor, mechanical ventilation, SOFA, and Charlson score). Heterogeneity: *I*^2^ = 0% (*Q* = 1.60, *p* = 0.45). *p < 0.05, *p < 0.01. Sensitivity analysis restricted to in-hospital mortality, pooled via fixed-effect meta-analysis. Adjusted for the same covariates as the primary model.

**Figure 4:**
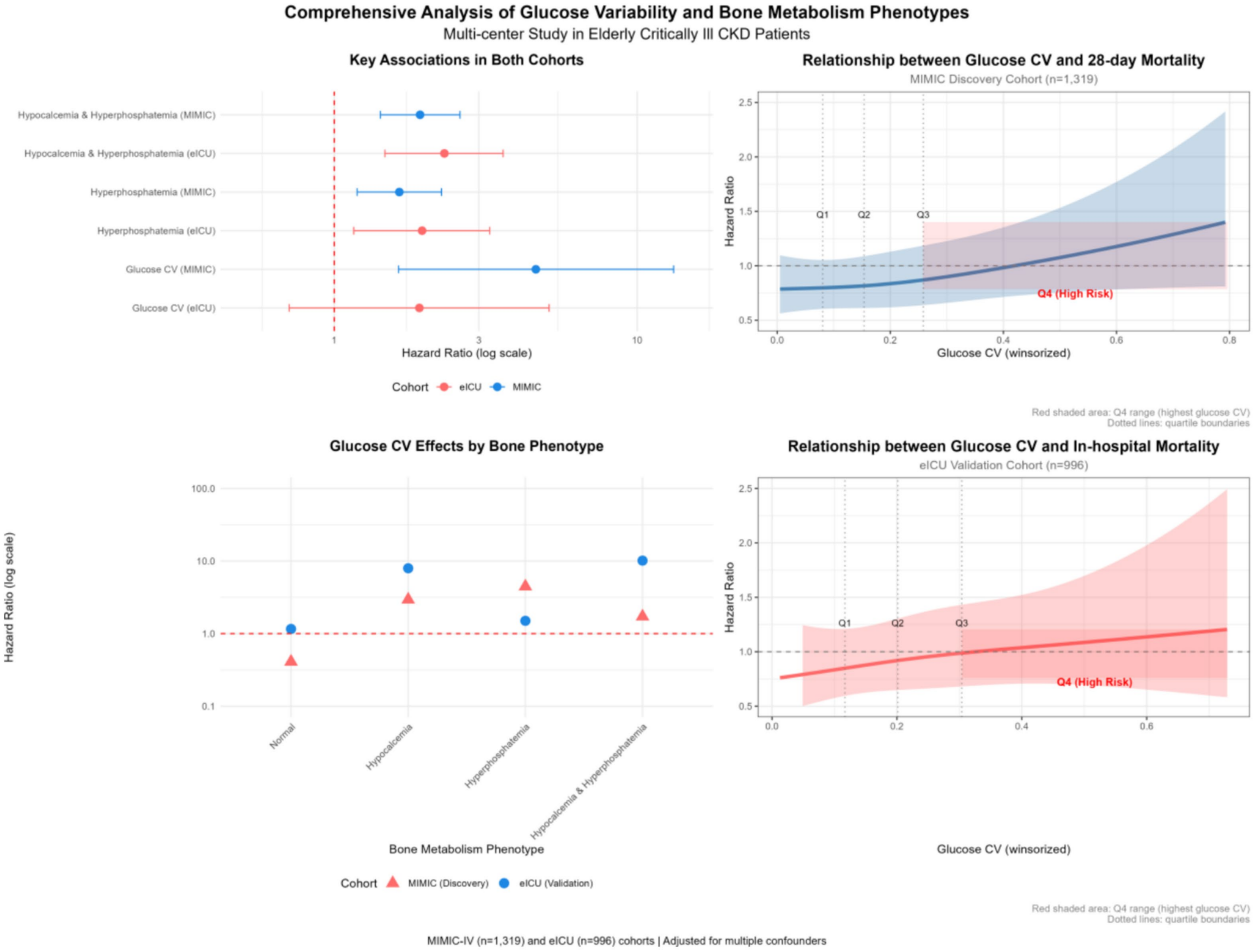
Associations of glucose CV and Calcium-phosphate phenotype subgroups with mortality across cohorts. (Upper left) Forest plot of Glucose CV (Q4 vs. Q1) and calcium-phosphate phenotypes. (Lower left) Stratified analysis of Glucose CV effect within each phenotype subgroup. (Upper right) RCS curve for Glucose CV and 28-day mortality in MIMIC-IV. (Lower right) RCS curve for Glucose CV and in-hospital mortality in eICU. Shaded areas represent 95% CIs.

Glucose variability (Glucose CV) was positively associated with mortality risk in three independent ICU cohorts (Table 3). In the discovery cohort MIMIC-IV, the highest quartile of GV (Q4 vs. Q1) significantly increased mortality risk (OR 1.36, 95% CI 1.05–1.77, *p* = 0.020), with a 7% increased risk per 0.1 unit increase in CV (OR 1.07, 95% CI 1.01–1.14, *p* = 0.050). The effect was stronger in the clinical validation cohort (Q4 vs. Q1: OR 4.09, 95% CI 1.45–12.8, *p* = 0.0105; per 0.1 unit: OR 1.23, 95% CI 1.00–1.51, *p* = 0.052). In the multicenter eICU validation cohort, Glucose CV showed an association consistent in direction with the single-center cohort (MIMIC-IV: HR 1.87, *p* = 0.040) (Figure 4, HR = 1.91, 95% CI 0.71–5.12, *p* = 0.197), but with a wider confidence interval, although statistical significance was limited by shorter follow-up time and number of events. The TyG index remained stable in eICU (HR = 1.26 (1.01–1.56), *p* = 0.040). Fixed-effects meta-analysis of the three cohorts confirmed the overall effect: Q4 vs. Q1 OR 1.44 (95% CI 1.15–1.82, *p* = 0.0018), and per 0.1 unit increase in CV OR 1.08 (95% CI 1.02–1.15, *p* = 0.008), indicating that despite baseline differences, the conditional effect after multivariate adjustment was highly consistent across the three cohorts (heterogeneity *I*^2^ = 0%, Q test *p* = 0.45, Supplementary Figure S2), confirming the robustness of the findings. To address potential heterogeneity in outcome definitions (28-day vs. in-hospital mortality), we performed a sensitivity meta-analysis restricted to in-hospital mortality (Table 3). The pooled OR was 1.47 (95% CI: 1.11–1.93, *p* = 0.0066), consistent with the primary meta-analysis (OR = 1.44, 95% CI: 1.15–1.82, *p* = 0.0018), confirming that the association is robust to outcome definition.

In the MIMIC-IV cohort, the combined hypocalcemia-hyperphosphatemia phenotype had the highest risk (Table 4): 1.92 (1.42–2.60, *p* < 0.001) at 28 days; HR 1.76 (1.6–2.26, *p* < 0.001) at 90 days. In the eICU cohort, the combined hypocalcemia-hyperphosphatemia phenotype also had the highest risk [Table 4, OR 2.31 (1.47–3.61, *p* < 0.001)]. Stratification by skeletal phenotypes revealed the strongest effect in the hypocalcemia-hyperphosphatemia group (HR 10.13, *p* = 0.001) (bottom left of Figure 4). In the actual clinical cohort, after multivariate adjustment, hyperphosphatemia [OR 2.36 (1.52–3.69, *p* < 0.001)] and hypocalcemia-hyperphosphatemia [OR 3.52 (2.35–5.34, *p* < 0.001)] were significantly and independently associated with mortality risk.

**Table 4:** Bone mineral phenotype associations across cohorts.

**Phenotype** **(vs Normal)**	**MIMIC-IV 28-day**		**MIMIC-IV 90-day**		**eICU-CRD in-hospital**		**Clinical in-hospital**	
	**HR** **(95% CI)**	***p* value**	**HR** **(95% CI)**	***p* value**	**OR** **(95% CI)**	***p* value**	**OR** **(95% CI)**	***p* value**
Hypocalcemia	1.01 (0.68–1.50)	0.96	1.05 (0.76–1.45)	0.78	1.77 (1.13–2.78)	0.013*	1.49 (0.94–2.39)	0.094
Hyperphosphatemia	1.64 (1.19–2.26)	0.003**	1.49 (1.14–1.95)	0.004**	1.95(1.16–3.26)	0.011*	2.36 (1.52–3.69)	<0.001***
Combined	1.92(1.42–2.60)	<0.001***	1.76 (1.36–2.26)	<0.001***	2.31 (1.47–3.61)	<0.001***	3.52 (2.35–5.34)	<0.001***

*p < 0.05, **p < 0.01, ***p < 0.001. MIMIC-IV: Multivariable Cox models (HR). eICU-CRD, Clinical: Multivariable logistic regression (OR). All adjusted for age, sex, severity scores, Charlson score, diabetes, insulin use, mechanical ventilation.

Analysis of secondary endpoints (Table 5) showed that Glucose CV had limited utility in predicting cardiorenal outcomes, but when combined with calcium-phosphate phenotype, it could predict 7-day new-onset RRT (OR 4.51, 2.49–8.18, *p* < 0.001), shock lasting >48 h (OR 2.39, 45–3.93, *p* = 0.001), and composite outcomes (OR 6.99, 4.07–12.01, *p* < 0.001).

**Table 5:** Secondary clinical outcomes (MIMIC-IV).

**Outcome**	**Glucose CV**		**Bone combined phenotype**	
	**OR (95% CI)**	***p* value**	**OR (95% CI)**	***p* value**
7-day RRT (16.8%)	0.71 (0.24–2.07)	0.53	4.51(2.49–8.18)	<0.001***
Shock >48 h (18.9%)	1.73 (0.66–4.53)	0.27	2.39 (1.45–3.93)	0.001**
Composite 28d (44%)	1.65 (0.71–3.84)	0.24	6.99 (4.07–12.01)	<0.001***

RRT: Renal Replacement Therapy. *p < 0.05, **p < 0.01, ***p < 0.001.

**Figure 5:**
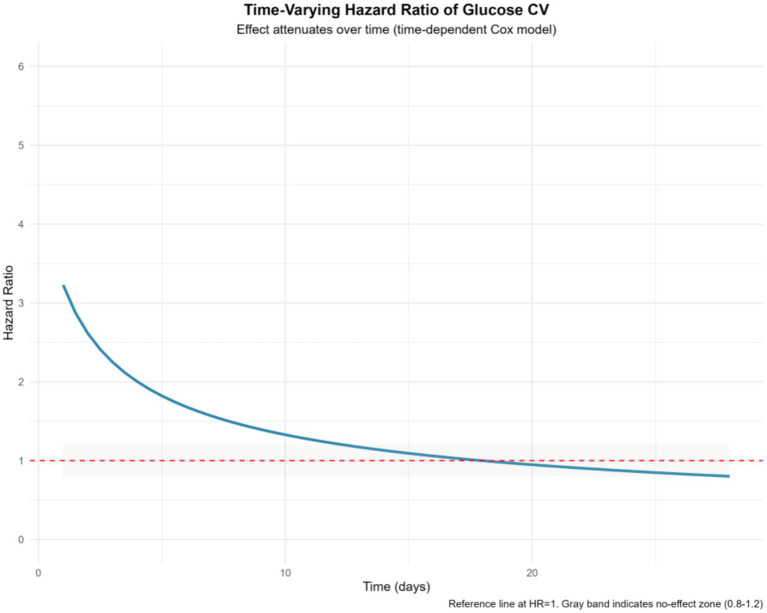
Time-varying effect curve (showing Glucose CV effect decaying over time). The solid line represents the hazard ratio for the effect of Glucose CV (per 0.1 unit increase) on 28-day mortality over time, estimated from a time-dependent Cox model adjusted for demographic and clinical covariates. The shaded area indicates the 95% confidence interval. The effect was highest on the day of ICU admission and gradually declined thereafter, becoming non-significant after approximately 14 days (time-interaction *p* = 0.065). The dotted horizontal line at HR = 1 indicates the null effect.

Several sensitivity analyses were performed to assess the robustness of our primary findings. First, a complete-case analysis restricted to patients with complete calcium data (*n* = 1,088, Table 6) yielded results consistent with the primary analysis (HR = 1.38, 95% CI 1.06–1.80, *p* = 0.015). Second, a time-dependent Cox model revealed that the effect of glucose variability on mortality was time-varying, with the strongest association observed in the first 14 days after ICU admission, after which the effect gradually attenuated (Figure 5). Third, in the MIMIC-IV cohort, adjustment for RRT within 7 days did not materially change the association (OR = 1.35 vs. 1.34 in the main model). Together, these sensitivity analyses confirm the robustness of our findings to different analytical approaches, including potential concerns related to assay detection limits.

**Table 6:** Sensitivity analyses in subsets (MIMIC-IV complete case, *n* = 1,088).

**Analysis**	**Model**	**28-day Mortality (377 events) OR/HR (95% CI)**	**28-day Mortality** **(377 events) *p* value**	**90-day Mortality (496 events) OR/HR (95% CI)**	**90-day Mortality** **(496 events) *p* value**
Glucose CV	Logistic (28d)	2.02 (0.86–4.73)	0.106	—	—
	Cox (90d)	—	—	1.88(1.07–3.30)	0.028*
Bone combined phenotype	Logistic (28d)	6.99 (4.07–12.0)	<0.001***	—	—
	Cox (90d)	—	—	1.91 (1.44–2.52)	<0.001***
Composite outcome (28d death/RRT, 479 events)					
Glucose CV	—	1.65 (0.71–3.84)	0.242	—	—
Bone Combined	—	6.99 (4.07–12.0)	<0.001***	—	—

*p < 0.05, **p < 0.01, ***p < 0.001.

## Discussion

Persistent hyperglycemia and insulin resistance inhibit osteoblast differentiation and bone formation while promoting osteoclast activity, leading to an imbalance in bone turnover, decreased bone mass, and deteriorating bone quality. High glucose can also cause abnormal collagen cross-linking in the bone matrix through increased oxidative stress and deposition of advanced glycation end products (AGEs) (12), resulting in fragile trabecular structure; even with normal bone mineral density(BMD), “brittle bones” may occur. Insulin itself is an anabolic hormone that promotes bone formation; insulin deficiency or resistance weakens the bone’s utilization of glucose and secretion of osteocalcin, forming a vicious cycle of “high blood sugar - low bone turnover” (13).

In this study, blood glucose variability (rather than merely average blood glucose) further represents acute “metabolic fluctuations.” In elderly critically ill CKD patients, severe fluctuations in blood glucose during the acute phase may amplify bone remodeling disorders through activation of stress responses, inflammatory pathways, and sympathetic nervous system excitation, manifesting as early high mortality risk (time-dependent Cox model: early HR = 3.84, *p* = 0.007).

In the eICU validation cohort, the association between high glucose variability and mortality was directionally consistent with the MIMIC-IV findings but did not reach statistical significance (OR = 1.44, 95% CI 0.83–2.49, *p* = 0.192). We attribute this to the shorter follow-up window of the eICU cohort (in-hospital mortality vs. 28-day mortality). In-hospital mortality captures only deaths occurring during the initial hospitalization, thereby censoring deaths that occur after discharge. The effect of glucose variability is likely time-accumulative—early glycemic fluctuations exert both acute and subacute effects that extend beyond the hospital stay. This interpretation is supported by the MIMIC-IV 90-day mortality analysis, which demonstrated a sustained effect of glucose variability (HR = 1.07 per 0.1unit CV), confirming its value in predicting medium-term (90-day) outcomes. The eICU non-significance thus likely reflects limited statistical power due to the truncated endpoint, rather than absence of a true biological effect.

Bone tissue is regarded as the “fourth glucose metabolic organ” after the liver, muscle, and fat, capable of secreting osteocalcin and other hormone-like factors that positively regulate insulin secretion and sensitivity (14). When bone mineral disorders such as hyperphosphatemia, hypocalcemia, and secondary hyperparathyroidism occur, the bone-derived endocrine function is impaired, leading to decreased osteocalcin secretion and disruption of the balance between bone formation and resorption, further exacerbating insulin resistance and difficulties in blood glucose control.

CKD-specific mechanisms include phosphorus retention, elevated FGF23 (15), and active vitamin D deficiency (16), which simultaneously damage bone structure and insulin signaling pathways, forming a “mutual drag” pattern between bone and glucose metabolism. Therefore, in elderly critically ill CKD patients, bone mineral disorders are not “independent complications” but directly participate in the pathological network of glucose metabolism imbalance and multi-organ failure.

Epidemiological studies show that patients with type 2 diabetes have an increased risk of fractures at the same or higher BMD levels (17), suggesting that “high glucose + abnormal bone remodeling” synergistically increases the risk of structural fragility and adverse outcomes after falls. In this cohort of critically ill CKD patients, increased blood glucose variability was significantly associated with mortality risk, while the combined low calcium-high phosphorus phenotype was also a strong risk factor for death; when both coexist, the risks of death, RRT demand, and persistent shock are significantly additive, reflecting the “synergistic attack” of glucose metabolic fluctuations and bone mineral disorders in critical conditions.

Acute inflammation and catecholamine surges induced by high GV (18) can inhibit the physiological regulation of bone remodeling by parathyroid hormone (PTH) (19), while severe bone mineral disorders leading to cardiovascular calcification further worsen hemodynamic stability (20), making blood glucose control more difficult (21).

Beyond calcium and phosphate homeostasis, bone-derived hormones such as FGF23 and PTH/PTHrP may play important roles in the acute metabolic crosstalk observed in our study. In CKD patients, circulating FGF23 levels increase progressively as renal function declines. Deregulated FGF23 has been linked to insulin resistance, pancreatic *β*-cell dysfunction, and systemic inflammation (22). Studies suggest FGF23 influences glucose metabolism via insulin signaling, oxidative stress, and inflammation. These maladaptive and diabetogenic responses may occur through direct effects on pancreatic *β*-cells as well as indirect effects via stimulation of pro-inflammatory factors (23). Elevated FGF23 levels have been associated with an increased risk of type 2 diabetes independent of traditional risk factors, and FGF23 is increasingly recognized as a key factor in the cardiovascular-kidney-metabolic (CKM) syndrome, connecting diabetes, CKD, and cardiovascular disease (22). PTH, in turn, has been shown to modulate pancreatic β-cell function and insulin secretion; chronic excess of PTH can impair insulin release (24). More recently, parathyroid hormone-related protein (PTHrP) has been identified as a key regulator of islet function, directly increasing β-cell proliferation and glucose-stimulated insulin secretion (Overexpression of PTHrP in β Cells Reveals Key Effects on Endoplasmic Reticulum Stress). While we did not measure these hormones directly in our study (a clear limitation), the observed additive risk of the ‘mineral disorder + high GV’ phenotype raises the hypothesis that bone-derived hormones—particularly FGF23 and PTH/PTHrP signaling—may mediate, at least in part, the bone–glucose crosstalk in critically ill CKD patients. Future prospective studies incorporating these biomarkers are warranted to elucidate the underlying mechanisms.

Single indicators are insufficient to capture truly high-risk populations. Relying solely on HbA1c or average blood glucose may miss patients with high GV but low averages; looking only at BMD or single electrolytes cannot reflect the true bone-mineral “phenotype” risk. Both glucose metabolism and mineral metabolism participate in the damage to the vascular, cardiac, renal, and immune systems, and when both systems are abnormal, the risks of death and organ failure often increase exponentially, rather than simply “adding one point each.” A comprehensive assessment is more likely to form actionable stratification strategies. By centering on blood glucose variability (such as CV, SD, or TIR) + bone mineral phenotype, a simple risk stratification model can be constructed to early identify “dual high-risk” patients for glucose and bone after ICU admission.

High-risk layers can adopt stricter blood glucose monitoring and variability control strategies (such as prioritizing continuous insulin pumps, reducing excessive glucose-lowering fluctuations), while actively correcting hyperphosphatemia, hypocalcemia, vitamin D deficiency, and metabolic acidosis, alleviating multi-organ impacts from both “glucose” and “bone” entry points. Some hypoglycemic agents (such as GLP-1RA, metformin, etc.) have been shown to have potential bone-protective or at least neutral effects in non-CKD populations (25), while some drugs (such as certain SGLT2i or thiazolidinediones) may increase fracture risk (26); a comprehensive glucose-bone risk assessment can help more reasonably weigh drug choices and nutritional supplementation plans in critically ill CKD patients.

Although the exposure windows differed between cohorts (24 h in MIMIC-IV vs. 48 h in eICU and Clinical cohorts), this heterogeneity reflects real-world variations in clinical practice. The consistently low statistical heterogeneity (*I*^2^ = 0% in primary meta-analyses) and the consistent sensitivity analysis (in-hospital mortality meta-analysis OR = 1.47, *p* = 0.0066) indicate that the observed association is robust to both exposure timing and outcome definition. This strengthens, rather than weakens, the generalizability of our findings. While heterogeneity in calcium measurement exists between cohorts (ionized calcium in MIMIC-IV and clinical cohorts vs. total calcium in eICU), this discrepancy is confined to the eICU cohort alone and was driven by data availability. The use of unadjusted total calcium in eICU introduces non-differential misclassification, biasing estimates towards the null. Despite this, we observed consistent associations across all cohorts, suggesting that the true biological effect is likely stronger than reported.

Based on our findings, we propose a pragmatic risk stratification scheme (Table 7): (1) patients with low GV (Q1–Q3) and normal mineral phenotype constitute the low-risk group; (2) patients with either high GV (Q4) or mineral disorder (hyperphosphatemia or combined phenotype) constitute the intermediate-risk group; and (3) patients with both high GV and mineral disorder constitute the high-risk group, warranting closer monitoring and potentially more intensive glycemic and metabolic management.

**Table 7:** Hypothesis-generating risk stratification framework.

Groups	**Normal phenotype**	**Abnormal phenotype**
Low GV (Q1–Q3)	Low risk	Intermediate risk
High GV (Q4)	Intermediate risk	High risk

GV, glycemic variability; Q1–Q3, lower three quartiles; Q4, highest quartile. Abnormal phenotype: hypocalcemia, hyperphosphatemia, or combined. This framework is derived from observational data and requires prospective validation.

The strengths of this study include the use of cohorts from two large independent ICU databases for development and validation, and verification in actual clinical ICU data. Although there are differences in specific data definitions, collection frequencies, and outcome indicators among several cohorts, the association between core metabolic disorders and adverse prognosis has been confirmed in both the eICU and clinical cohorts. This precisely demonstrates that our findings have good robustness across different clinical practice patterns and data processing methods.

The main limitation is the absence of long-term survival data beyond hospital discharge in both the eICU and clinical cohorts, which precluded 28-day or 90-day mortality analyses in these validation cohorts. While in-hospital mortality serves as an acceptable proxy for short-term outcomes in ICU studies, it may underestimate the full effect of glucose variability on longer-term prognosis. Future studies with extended follow-up are needed to confirm the sustained nature of these associations. Second, our assessment of glycemic variability relied on intermittent point-of-care glucose measurements rather than continuous glucose monitoring (CGM). While the coefficient of variation (CV) is the most widely used metric for quantifying GV in ICU populations and has been consistently associated with short-term mortality in large-scale meta-analyses—intermittent sampling inevitably underestimates true glycemic fluctuations and cannot capture time-in-range or hypoglycemia burden (27). Importantly, the accuracy and reliability of CGM in the ICU setting remain subjects of ongoing debate (28). Studies have shown that CGM accuracy in critically ill patients is lower than in outpatients (29), and may be suboptimal during the first 36 h of use, outside normoglycemic ranges, or during higher-dose vasopressor therapy (30). Future prospective studies incorporating CGM are warranted to validate our findings and to explore whether CGM-derived metrics provide additional prognostic value beyond CV. Third, detailed treatment variables were not uniformly captured across all cohorts. In the MIMIC-IV cohort, IV vs. SC insulin route was available for only 21.7% of insulin users; corticosteroid use (31.8%), nutritional support (23.9%), phosphate binders (29.3%), and vitamin D therapy (13.3%) had similarly limited coverage. While we adjusted for any insulin use, vasopressor use, mechanical ventilation, RRT within 7 days, and severity scores, we cannot exclude residual confounding by unmeasured treatment details. Future prospective studies with systematic collection of these variables are warranted. Although we used two large public databases (MIMIC-IV and eICU-CRD) for discovery and validation, the third cohort was derived from a single Chinese tertiary hospital, which may limit generalizability to other populations and healthcare settings. Differences in laboratory standards (ionized calcium in MIMIC-IV vs. total calcium in eICU and clinical cohorts) and clinical practices (e.g., insulin protocols, RRT initiation criteria) may have contributed to the observed heterogeneity. We have provided a detailed harmonization table (Supplementary Table S4) summarizing the laboratory assays, thresholds, and missing rates for each cohort. Future studies in diverse geographic and ethnic populations are needed to validate our findings.

The findings of this study generate several hypotheses for future investigation. First, prospective cohort studies with standardized collection of glucose metrics (including continuous glucose monitoring and time-in-range) and bone-mineral biomarkers (PTH, FGF23, vitamin D) are needed to validate the ‘double-hit’ phenotype in real-time clinical settings. Second, randomized controlled trials evaluating glucose stabilization strategies (e.g., continuous insulin infusion vs. standard sliding-scale insulin) in high-risk patients (high GV + abnormal calcium-phosphate phenotype) are warranted to determine whether reducing GV improves clinical outcomes. Third, mechanistic studies are needed to elucidate the crosstalk between bone-derived hormones (e.g., FGF23, osteocalcin) and insulin signaling pathways, as well as the role of inflammation in linking glucose fluctuations to acute kidney injury and cardiovascular events in CKD patients.

## Conclusion

Bone mineral metabolism disorders are a stable independent predictor of mortality in elderly critically ill CKD patients, fully validated in multi-center cohorts; blood glucose variability shows time-varying mortality risk effects, clearly defined in the discovery cohort, with consistent trends in the validation cohort but not reaching statistical significance. It is recommended to integrate personalized risk management of blood glucose variability and mineral metabolism markers.

## Data Availability

The datasets presented in this study can be found in online repositories. The names of the repository/repositories and accession number(s) can be found in the article/Supplementary material.
