# Supplementary material for: Combined impact of glucose variability and mineral disorders on mortality and severe cardiorenal events in critically ill CKD patients: a multicenter cohort study
Source: Front Med (Lausanne). 2026 Jul 8;13:1826900. doi: 10.3389/fmed.2026.1826900 (PMC13388537; doi:10.3389/fmed.2026.1826900)
Supplement: Supplementary file 1 [file Supplementary_file_1.docx]

**Supplementary Material**

**Figure S1 Baseline Heterogeneity Between Cohorts (SMD ≥0.3 = Moderate/High)**


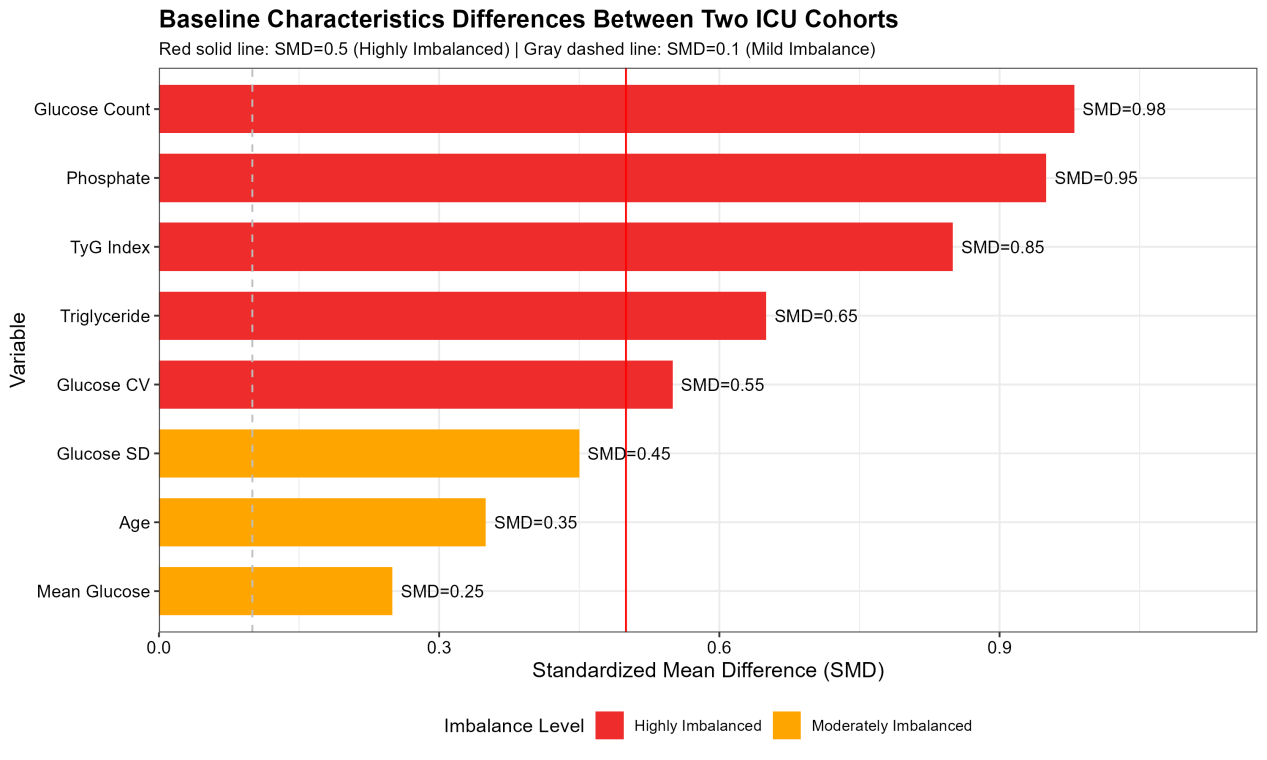


Standardized mean differences (SMDs) between cohorts. Laboratory measurements showed largest differences, reflecting protocol variations.SMD calculated using pooled standard deviation method.

**Figure S2 Meta-analysis of MIMIC-IV, eICU and clinical cohort**


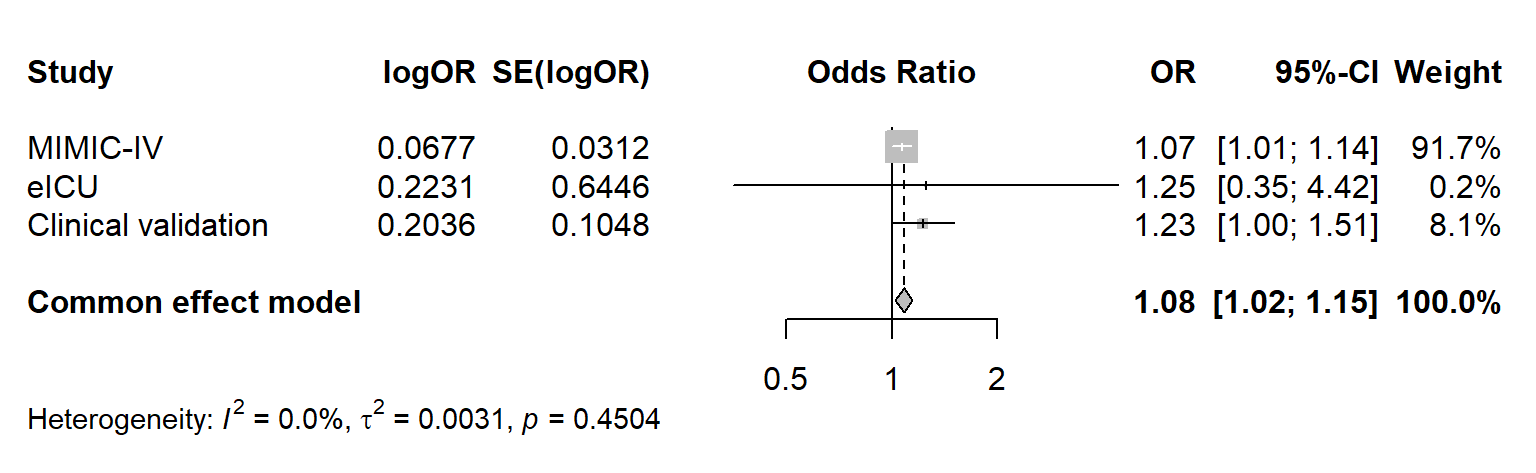


Random-effects meta-analysis of the association between glucose coefficient of variation and mortality in the MIMIC-IV (discovery) , eICU (validation) and clinical(validation) cohorts. There was no significant heterogeneity between cohorts (I² = 0%).

**Table S1：Detailed results of the Cox model with time-dependent covariates (including time interaction terms)**

| Quartile | N | Deaths | Mortality Rate |
| --- | --- | --- | --- |
| Q1 | 330 | 109 | 33.00% |
| Q2 | 330 | 108 | 32.70% |
| Q3 | 329 | 106 | 32.20% |
| Q4 | 330 | 132 | 40.00% |

The absolute risk difference between Q4 and Q1 was 7.0 percentage points, corresponding to a number needed to harm (NNH) of approximately 14.

**Table S2：Detailed results of the Cox model with time-dependent covariates (including time interaction terms)**

| **Model** | **Variable** | **Coefficient** | **HR** | **SE** | **95%CI** | **P value** |
| --- | --- | --- | --- | --- | --- | --- |
| 28-Day Mortality | Glucose CV | 1.5329 | 4.632 | 0.5343 | 1.625-13.199 | 0.0041 |
| 28-Day Mortality | tt(Glucose CV) | -0.5209 | 0.594 | 0.2724 | 0.348-1.013 | 0.0558 |
| 28-Day Mortality | TyG | 0.2757 | 1.317 | 0.0488 | 1.197-1.45 | 0 |
| 28-Day Mortality | tt(TyG) | -0.109 | 0.897 | 0.0246 | 0.854-0.941 | 0 |
| 28-Day Mortality | bone_phenotype_Hyperphosphatemia | 0.4952 | 1.641 | 0.164 | 1.19-2.263 | 0.0025 |
| 28-Day Mortality | bone_phenotype_Hypocalcemia & Hyperphosphatemia | 0.652 | 1.919 | 0.1539 | 1.42-2.595 | 0 |
| 90-Day Mortality | Glucose CV | 1.3449 | 3.838 | 0.4961 | 1.452-10.146 | 0.0067 |
| 90-Day Mortality | tt(Glucose CV) | -0.3623 | 0.696 | 0.196 | 0.474-1.022 | 0.0646 |
| 90-Day Mortality | TyG | 0.2403 | 1.272 | 0.0451 | 1.164-1.389 | 0 |
| 90-Day Mortality | tt(TyG) | -0.071 | 0.931 | 0.018 | 0.899-0.965 | 0.0001 |
| 90-Day Mortality | bone_phenotype_Hyperphosphatemia | 0.3997 | 1.491 | 0.1375 | 1.139-1.953 | 0.0037 |
| 90-Day Mortality | bone_phenotype_Hypocalcemia & Hyperphosphatemia | 0.5581 | 1.747 | 0.1288 | 1.358-2.249 | 0 |

**Table S3：Proportional Hazards Assumption Testing and Model Solutions**

| **Variable** | **Standard Cox P** | **Stratified Cox P** | **Time-Dependent Cox** | **Solution** | **Notes** |
| --- | --- | --- | --- | --- | --- |
| **Glucose CV** | 0.004 | 0.042 | Pass | Time interaction | Strong early effect |
| **TyG Index** | <0.001 | 0.000047 | Pass | Time interaction | Time-varying effect |
| Age | 0.152 | 0.152 | Pass | None | PH met |
| Gender | 0.033 | NA | Pass | Stratified | PH violated |
| SOFA Score | 0.59 | 0.59 | Pass | None | PH met |
| Charlson Score | 0.527 | 0.527 | Pass | None | PH met |
| **Insulin Use** | 0.0076 | NA | Pass | Stratified | PH violated |
| **Mech. Vent**. | 0.0019 | NA | Pass | Stratified | PH violated |
| Bone Phenotype | 0.296 | 0.296 | Pass | None | PH met |
| **Global Test** | <0.001 | <0.001 | Pass | Multiple | Fully addressed |

NA: Stratified variable, PH test not applicable

Bold: Variables violating PH assumption (P<0.05)

**Table S4:** **Laboratory assays and thresholds for calcium measurement across cohorts**

| **Parameter** | **MIMIC-IV** | **eICU-CRD** | **Clinical Cohort** |
| --- | --- | --- | --- |
| Calcium assay | Ionized calcium | Total calcium | Ionized calcium |
| Reference range | 1.12–1.30 mmol/L | 2.10–2.60 mmol/L | 1.12–1.30 mmol/L |
| Abnormal threshold | <1.12 mmol/L | <2.10 mmol/L | <1.12 mmol/L |

This table summarizes the differences in calcium measurement methods and abnormality thresholds across the three cohorts. The MIMIC-IV and clinical cohorts used ionized calcium, whereas the eICU cohort used total calcium due to the limited availability of ionized calcium measurements (27.8% coverage). Reference ranges were based on each institution’s laboratory standards. These differences represent a limitation of multi‑database studies and are addressed in the Discussion.
